# Single Chain Variable Fragments Produced in *Escherichia coli* against Heat-Labile and Heat-Stable Toxins from Enterotoxigenic *E*. *coli*

**DOI:** 10.1371/journal.pone.0131484

**Published:** 2015-07-08

**Authors:** Christiane Y. Ozaki, Caio R. F. Silveira, Fernanda B. Andrade, Roberto Nepomuceno, Anderson Silva, Danielle D. Munhoz, Bruno B. Yamamoto, Daniela Luz, Patrícia A. E. Abreu, Denise S. P. Q. Horton, Waldir P. Elias, Oscar H. P. Ramos, Roxane M. F. Piazza

**Affiliations:** 1 Laboratório de Bacteriologia, Instituto Butantan, São Paulo, SP, Brasil; 2 CEA, iBiTecs, SIMOPRO, Gif sur Yvette, France; Institute Pasteur, FRANCE

## Abstract

**Background:**

Diarrhea is a prevalent pathological condition frequently associated to the colonization of the small intestine by enterotoxigenic *Escherichia coli* (ETEC) strains, known to be endemic in developing countries. These strains can produce two enterotoxins associated with the manifestation of clinical symptoms that can be used to detect these pathogens. Although several detection tests have been developed, minimally equipped laboratories are still in need of simple and cost-effective methods. With the aim to contribute to the development of such diagnostic approaches, we describe here two mouse hybridoma-derived single chain fragment variable (scFv) that were produced in *E*. *coli* against enterotoxins of ETEC strains.

**Methods and Findings:**

Recombinant scFv were developed against ETEC heat-labile toxin (LT) and heat-stable toxin (ST), from previously isolated hybridoma clones. This work reports their design, construction, molecular and functional characterization against LT and ST toxins. Both antibody fragments were able to recognize the cell-interacting toxins by immunofluorescence, the purified toxins by ELISA and also LT-, ST- and LT/ST-producing ETEC strains.

**Conclusion:**

The developed recombinant scFvs against LT and ST constitute promising starting point for simple and cost-effective ETEC diagnosis.

## Introduction

Up to 5 million cases of diarrhea are reported around the world leading to thousands of deaths per year in children under five years of age [1]. Diarrheagenic *Escherichia coli* (DEC) are the most frequent bacterial etiological agent, in particular, enterotoxigenic *E*. *coli* (ETEC), which is endemic in essentially all developing countries. Also, approximately 20 to 60% of travelers to developing countries contract diarrheal disorders being ETEC the etiological agent responsible for most of them [2]. ETEC strains produce colonization factors, which allow the organisms to readily colonize the small intestine and in this way leading to diarrhea due to the production of heat-labile (LT) and/or heat-stable (ST) enterotoxins [3, 4, 5]. Since ETEC comprise a wide range of O antigenic types, diagnosis must depend upon the detection of LT and ST enterotoxins.

As revised and well addressed by Qadri and colleagues several immunoserological assays were established for the detection of ST and LT, but regrettably, in developing countries there are still no simple, readily available tools and/or methods that can be used to identify these organisms in minimally equipped laboratories [6]. Usually, serotyping-based diagnosis is the only methodology available in limited-resources settings, employing either commercial or in house antisera [7]. For that reason, many laboratories conducting studies on the etiology of diarrhea in developing countries do not include ETEC in their routine diagnostic, and only research or reference laboratories are skilled to identify these bacteria [6, 7].

Monoclonal antibodies began to be produced *in vitro*, showing homogeneity and specificity, characteristics not observed in polyclonal antibodies [8]. Even with desirable characteristics, both for diagnosis and therapy, the production of monoclonal antibodies have limitations, such as high cost and long culture periods. To circumvent such limitations, genetic engineering has been used to obtain recombinant antibodies that maintain or improve the functional properties of an antibody.

Among the different formats of antibodies fragments, single chain variable fragment or scFv is one of the most popular due to its versatility. Presenting molecular mass between 27 and 30 kDa, the scFv comprises heavy (VH) and light (VL) chain variable domains, connected by a flexible polypeptide (linker). The insertion of a polypeptide linker allows the correct distance between C-terminal and N-terminal domains, preserving molecule flexibility to a better antigen binding, and preventing their dissociation. Generally, polypeptides of 15 aminoacids length with (Gly_4_Ser)_3_ in its composition are the most used, once linkers with length greater than 12 aminoacid residues allow VH and VL interactions similar to native [9, 10, 11, 12, 13, 14]. Among the expression systems available for the antibody fragments production, *E*. *coli* has been widely used, presenting various advantages such as easy handling, fast growth, short time for protein expression, simple and inexpensive culture media, and high performance. Another factor that contributes to their broad range use is the availability of a large number of vectors and strains, which facilitates the gene cloning and the proteins production [15, 16].

The convenience of genetic engineering has enabled the development of recombinant antibodies in scFv format against different antigens of DEC pathotypes that can be used as a tool for diagnosis. Considering that, the objective of this work consisted in the production and characterization of scFv molecules to detect LT and ST toxins of ETEC.

## Materials and Methods

### Ethics statement

No animal model was employed in the present work. The hybridomas used as template for scFv development were previously obtained [17, 18] for LT monoclonal antibody (mAb) and for ST mAb, respectively. All experiments were conducted in agreement with the Ethical Principles in Animal Research, adopted by the Brazilian College of Animal Experimentation, and they were approved by the Ethical Committee for Animal Research of Butantan Institute (314/06). Y-1 cells, from mouse adrenal gland (ATCCCCL79), and Caco-2, from human colorectal adenocarcinoma (ATCCHTB37), were used in LT and ST cell interaction assays, respectively.

### Bacterial strains and plasmids

The following K12 *E*. *coli* strains were used: DH5α (Stratagene, USA), BL21 (DE3) (Novagen, USA) and C43 (DE3) (Lucigen, USA). The plasmid vector pET28a was obtained from Novagen (USA) and the pGEM-T Easy Vector System kit from Promega (USA). Bacterial isolates used in this study consisted of strains previously defined as ETEC by the presence of LT and/or ST encoding-gene, as well as the production of the respective toxins [18]. Also, ETEC H10407 (O78:H11) and 3321–4 (O153:H45) were employed as ST/LT-producing and ST-producing prototypes, respectively [19, 20].

### PCR analyses for toxins types

#### Primer design

Alignment of multiple available sequences of *eltA* (LTI) from GenBank (NC_014232, FN649417.1, AP010910, NC_017722) was employed to determine the conserved region of this gene and used to design the following primers sequences: (F) 5´-GGCGACAAATTATACCGTGC-3´ and (R) 5´-GCCGGTTTGTGTTCCTCTC-3´. The primers sequences used to amplify *stIa* (STp) [(F) 5´-TTTCCCCTCTTTTAGTCAGTCAA-3´ and (R) 5´-GCAGGATTACAACACAATTCACAGCAG-3´] and *stIb* (STh) [(F) 5´-TGCTAAACCAGTAGAGTCTTCAAAA-3´ and (R) 5´-GCAGGATTACAACACAATTCACAGCAG-3´] have been described elsewhere [21].

#### DNA isolation

Bacterial DNA was obtained by boiling method. Briefly, the isolates were cultivated in Luria-Bertani (LB) broth (Difco, USA) overnight at 37°C. One mL from this culture were centrifuged to 5,500 x g for 2 min, and then washed in the same volume with sterile ultrapure water and centrifuged again. Pellet was resuspended in 100 μL of sterile ultrapure water, boiled at 100°C for 10 min and centrifuged to 2,200 x *g* for 5 min. The supernatants were used as templates in PCR for detection of *eltA* gene (94°C– 1 min, 94°C– 1 min / 60°C– 1 min / 72°C– 1 min (35X), 72°C– 7 min, 4°C) and *st* genes subtypes (98°C– 50 s, 95°C– 30 s / 60°C– 30 s / 72°C– 30 s (30X), 72°C– 7 min, 4°C). Amplification reactions were performed on Veriti 96-well thermal cycler (Applied Biosystems, USA), with mixtures of 25 μL containing 2 μL of bacterial lysates, 0.4 mM dNTPs, 1X PCR buffer, 2 mM MgCl_2_, 0.5 mM of each primer and 1 U of *Taq* DNA Polymerase (Invitrogen, USA). PCR products were analyzed by 3% agarose gel electrophoresis after staining with GelRed (Biotium, USA)

### Toxins

The purified LT toxin [22] was kindly donated by Dr. John Clements from the Department of Microbiology and Immunology, Tulane University Health Sciences Center, New Orleans. ST toxin was obtained as described elsewhere [18].

### Amplification of heavy and light chains variable domains from hybridomas producing anti-LT and anti-ST monoclonal antibodies

Total RNA from 6x10^5^cells/mL from hybridomas producing anti-LT and anti-ST monoclonal antibodies, previously described [17, 18], were extracted by RNeasy Mini Kit (QIAGEN, Germany) following manufacturer recommendations, and 1 μg was reverse transcribed using random hexamer primers by using first-strand cDNA Synthesis kit (GE Healthcare, USA). DNA fragments corresponding to heavy and light chains variable domains were obtained by PCR amplification using Mouse ScFv Module of Recombinant Phage Antibody System kit (GE Healthcare, USA), cloned into pGEM-T Easy Vector (Promega, USA) and sequenced using M13 and SP6 sequencing primers, on ABI 3730 DNA Analyser (Life Technologies, USA). The obtained sequences were analyzed using BLAST and both scFv-LT and scFv-ST were designed by joining coding sequences of heavy-chain variable domain (VH) and light-chain variable domain (VL) with an intermediary flexible linker (Gly_4_Ser)_3_, in the VH-linker-VL orientation. Based on designed sequences, optimized genes were synthesized for expression in *E*. *coli* (GeneArt, Germany).

### Subcloning of scFv-LT and scFv-ST genes into pET-28a expression vector

Forward and reverse primers including *BamHI* and *XhoI* (underlined), respectively, were designed in order to allow the subcloning of these synthetic genes:

- scFv-LT Forward (5'-GGATCCGTGAAACTGCAGGAAAGCG-3'),
- scFv-LT Reverse (5'-CTCGAGTCATTTCAGTTCCAGTTTGGTGC-3'),
- scFv-ST Forward (5’-GGATCCGTGAAACTGCAGCAGAGCG-3’) and
- scFv-ST Reverse (5’-CTCGAGTCATTTAATTTCCAGTTTGGTGCC-3’)

Both, scFv-LT and scFv-ST genes, were PCR amplified using 0.2 mM dNTPs, 1.5 mM MgCl_2_, 0.2 mM of each primer, 2.5 U Pfx DNA Polymerase (Invitrogen EUA), 50 ng plasmid DNA containing the synthetic gene, PCR buffer, and ultrapure water to a total volume of 50 μL. Water was used as negative control. The reactions were performed in Gene Amp PCR System 9700 (Applied Biosystems, EUA) using 35 cycles of: 94°C for 50 sec, 58°C for 50 sec and 68°C for 1 min. The PCR products of expected size were identified by agarose gel (1.0%) electrophoresis and isolated with PureLink Quick Gel Extraction Kit (Invitrogen, USA) according to manufacturer recommendations.

Terminal adenines were added to purified products by DNA polymerase treatment, the fragments were cloned into pGEM-T Easy vector and resulting vectors were digested with *BamHI* and *XhoI*. Digested fragments were subcloned into pET-28a (Novagen, USA), independently, using 25 ng of each fragment, 50 ng of pET-28a vector, ligation buffer, 40 U of T4 DNA ligase (New England Biolabs, USA), and sterile ultrapure water to a final volume of 10 μL. The reactions were incubated in GeneAmp PCR System 9700 thermal cycler (Applied Biosystems, USA) at 16°C for 18 h. The total volume of reactions was used to transform chemical competent *E*. *coli* DH5α cells, and selected transformants were tested for fragments presence by restriction analyses and sequencing using M13 and SP6 sequencing primers. Once confirmed, recombinant pET-scFv-LT and pET-scFv-ST were used to transform C43 (DE3) and BL21 (DE3) *E*. *coli* cells, respectively.

### Expression of scFv-LT and scFv-ST recombinant antibodies

For scFv-LT recombinant antibody expression, colonies obtained after *E*. *coli* C43 (DE3) transformation were pre-cultured in 10 mL of LB medium containing kanamycin 50 μg/mL (Invitrogen, USA) at 37°C, under stirring conditions (200 rpm) for 18 h (C25 Incubator Shaker NewBrunswick Scientific). Overnight culture was used to inoculate 500 mL fresh LB medium at 1:50 dilution. To induce scFv-LT expression, a final concentration of 1 mM isopropyl-β-D-thiogalactopyranoside (IPTG) (Sigma-Aldrich, USA) was added to the culture at an optical density interval (600 nm wavelength) of 0.6–0.8 (Spectronic 20—Genesys). The culture was incubated at 37°C, under stirring conditions (200 rpm) for 3 h before centrifugation at 5,000 x *g* for 10 min. Aliquots of 1 mL were collected before and 3 h after induction. The same conditions were employed for scFv-ST antibody expression, except that *E*. *coli* BL21 (DE3) cells were cultivated in 2-YT medium containing kanamycin (50 μg/mL) (Invitrogen, USA) for 4 h incubation after IPTG addition [23].

### Inclusion bodies isolation and solubilization

Isolation of inclusion bodies was performed according to Lamberski et al [24], with modifications. Briefly, bacterial pellet was ressuspended in 30 mL of lysis buffer (20 mM sodium phosphate, 500 mM sodium chloride and 5mM imidazole, pH 7.4), and submitted to bacterial disruption under pressure of 2,000 psi, using French Pressure (Thermo Scientific, USA). Subsequently, the lysate was centrifuged at 10,000 x *g* for 20 min at 20°C (Eppendorf 5804-R), and the insoluble fractions were ressuspended in 20 mL lysis buffer plus 10% Triton X-100, incubated for 10 min on ice and centrifuged at 15,000 x *g* for 15 min, 4°C (Eppendorf). This step was repeated once with lysis buffer plus 1% Triton X-100, and after centrifugation, the pellet was ressuspended in 20 mL lysis buffer and centrifuged again at 15,000 x *g*, 4°C for 15 min. For scFv-LT purification, inclusion bodies were solubilized with 20 mL of solubilizing buffer (20 mM sodium phosphate, 500 mM sodium chloride, 8 M urea, 5 mM imidazole, pH 7.4) at room temperature for 18 h. For scFv-ST the inclusion bodies were solubilized with 50 mM Tris-HCl, 200 mM NaCl, 8 M urea and 5 mM imidazole, pH 7.5 under stirring conditions at 4°C for 18 h. After solubilization both recombinant antibodies were centrifuged at 12,000 x *g* for 30 min and the supernatant was filtered through a 0.45 μm membrane before purification.

### Purification of scFv-LT and scFv-ST recombinant antibodies

Both scFv-LT and scFv-ST were purified as follow: the filtrates were subjected to affinity chromatography using a pre-packed Nickel-Sepharose HisTrap HP 5 mL column (GE Healthcare, UK), previously equilibrated with 25 mL of 50 mM of Tris-HCl (pH 7.5) containing 200 mM of NaCl and 5mM imidazole in AKTA prime plus (GE Healthcare, UK). After washing, the samples were eluted with gradient buffer from 0 to 100% of 50 mM Tris-HCl (pH 7.5) containing 200 mM NaCl, 500 mM imidazole and 8 M urea. All purification steps were analyzed by SDS-PAGE [25].

### Recombinant antibody scFv-LT and scFv-ST refolding

Following purification, the process of recombinant antibodies refolding was performed according to Tsumoto et al and Umetsu et al [26, 27], with modifications. Briefly, scFv-LT recombinant antibodies were subjected to reduction of disulfide bonds by addition of 10 mM 2-mercaptoethanol (Sigma-Aldrich, USA) and maintained at room temperature under gentle shaking for 1 h. To remove the reducing agent, denatured recombinant antibody was dialyzed against 20 mM sodium phosphate, 500 mM sodium chloride, 8 M urea, pH 7.4 for 18 h at 4°C with gentle stirring. After that, a stepwise dialysis was performed in same buffer containing decreasing urea concentration (6 M, 4 M, 2 M, 1 M and 0.5 M) for 12 h at 4°C with gentle stirring. In the two-dialysis steps (1 M and 0.5 M urea), 400 mM L-arginine (Sigma-Aldrich, USA) and 375 μM of oxidized glutathione (GSSG) (USB, EUA) were added. Final dialysis was performed in buffer without urea, for 18 h at 4°C with gentle stirring. Same conditions were employed for scFv-ST refolding, except that, in the last step (0.5 M urea) only 375 mM of oxidized glutathione was added. Final dialysis steps were done in absence of urea. Both proteins were concentrated in PEG 6000, quantified by Micro BCA Protein Assay kit (Thermo Scientific—USA) and analyzed by SDS-PAGE after Coomassie blue staining [25].

### Functional analysis of recombinants antibodies scFv-LT and scFv-ST

Employing different immunoassays, the function of scFv-LT and scFv-ST were probed using their respective purified toxins or LT-, ST- and ST/LT-producing ETEC [18]. For scFv-LT test by immunoblotting, 15 μg of purified LT toxin was separated by SDS-PAGE 15% [25] and transferred to nitrocellulose membrane [28]. The membrane strips were blocked with 5% skimmed milk solution at room temperature for 1 h and incubated with 200 μg/mL of scFv-LT at 4°C for 18 h followed by incubation with an IgG2a monoclonal anti-His antibody (1:3,000) (GE Healthcare, USA) and a goat IgG anti-mouse peroxidase-conjugated (1:1,000) (Zymed, USA). Both incubations were done at room temperature for 1 h. The reaction was developed using 3,3'-diaminobenzidine (DAB) in the presence of H_2_O_2_ (Promega Corporation, Madison, WI, USA) and stopped with distilled water. Monoclonal anti-LT antibody (50 μg/mL) was used as positive control, incubated with the membrane followed by a goat IgG anti-mouse peroxidase-conjugated incubation (1:1,000) (Zymed, USA).

The immunoblotting test for ST, three μg of ST was separated by 10% tris-tricine SDS-PAGE electrophoresis under reducing conditions [29] and transferred to a PVDF membrane (Amersham Biosciences, Little Chalfont, UK) at 150 mA, 4°C for 18 h. The membrane was incubated with 30 μg/mL of scFv-ST followed by monoclonal anti-His antibody and a goat IgG anti-mouse peroxidase-conjugated as described above for scFv-LT immunoblotting. Monoclonal anti-ST antibody (30 μg/mL) was used as positive assay control. Development of the assay was the same as described for scFv-LT.

#### Immunofluorescence analysis

For scFv-LT, immunofluorescence assay was performed as described by Dorsey et al [30]. LT toxin and Y-1 adrenal cells were incubated at 37°C for 7 h using 2.5 μg/mL of LT purified toxin. ScFv-LT (200 μg/mL) diluted in blocking buffer (1% BSA in 10 mM PBS) was added and incubated (37°C, 1 h), followed by incubation of monoclonal IgG2a anti-His (1:2,000) (GE Healthcare, USA) and anti-mouse IgG-FITC (1:100), both diluted in blocking solution and incubated at room temperature, for 1 h. After each incubation period the cells were washed three times during 5 min employing 0.01 M phosphate buffer saline pH 7.2 (PBS) containing 1% bovine serum albumin (BSA). Monoclonal anti-LT antibody (200 μg/mL) and anti-mouse IgG-FITC (1:100) were used as control. The reactions were visualized on Axioskop fluorescence microscope (Zeiss, Germany), with a 400X magnification. The reactions control used Y-1cells with either scFv-LT or MAb anti-LT plus anti-mouse IgG-FITC in the absence of toxin.

For detection of ST toxin using scFv-ST, Caco-2 cell line was used following the protocol described by Buc et al [31]. Caco-2 cells were incubated (37°C, 7 h) in the presence of ST toxin (2.5 μg/mL). scFv-ST (100 μg/mL) diluted in blocking buffer was added and incubated (37°C for 1 h), followed by the same protocol as described for scFv-LT detection. Monoclonal anti-ST antibody (100 μg/mL) was employed as positive assay control. All conditions adopted were the same as described for scFv-LT.

#### Capture ELISA for detection of biological activity of recombinant antibody scFv-LT

The ability of recombinant antibody scFv-LT to recognize LT toxin produced by ETEC isolates was tested by a capture ELISA, essentially as described by Rocha et al [18]. Briefly, after incubation with supernatants of ETEC isolates, 30 μg/mL of recombinant antibody scFv-LT was employed followed by an IgG2a monoclonal anti-His antibody (GE Healthcare, USA) 1:6,000 dilution, incubated for 30 minutes at 37°C. Goat anti-mouse IgG peroxidase-conjugate (Sigma-Aldrich, USA) was used at a dilution of 1:2000 in blocking solution (5% skimmed milk in PBS 10 mM), and the reaction was developed with a chromogenic solution containing 0.5 mg/mL o-phenylenediamine (OPD) (Sigma-Aldrich, USA). The absorbance was measured at wavelength of 492 nm in a Multiskan EX ELISA reader (Labsystems, Milford, MA, USA). Monoclonal anti-LT (18.5 μg/mL) was used as positive control to detect LT in ETEC H10407 prototype strain.

#### Indirect ELISA

The recombinant antibody scFv-ST was used to recognize ST toxin produced by ETEC isolates by indirect ELISA, as described by Rocha et al [18]. ELISA plates were coated with ETEC isolates supernatants and, after incubation, 2.5 μg/mL of recombinant antibody scFv-ST was added. The same antibodies conjugates described for scFv-LT was employed to detect antigen-antibody reaction. As positive control, 20 μg/mL of monoclonal anti-ST were used to detect ST in ETEC 3321–4 prototype strain.

## Results

### Toxins typing of ETEC isolates

The LT-, ST- and ST/LT-producing ETEC strains employed in this study were subtyped by PCR. PCR generates the following amplicons: 150 bp for *eltA* gene, 159 bp for *stIa* and 138 bp for *stIb*. These analyzes showed that all LT-producing strains were typed as LTI and among ST-producing strains, four were STp (STIa) and 37 were STh (STIb) strains.

### Single chain fragment variable (scFv) construction

**Fig 1:**
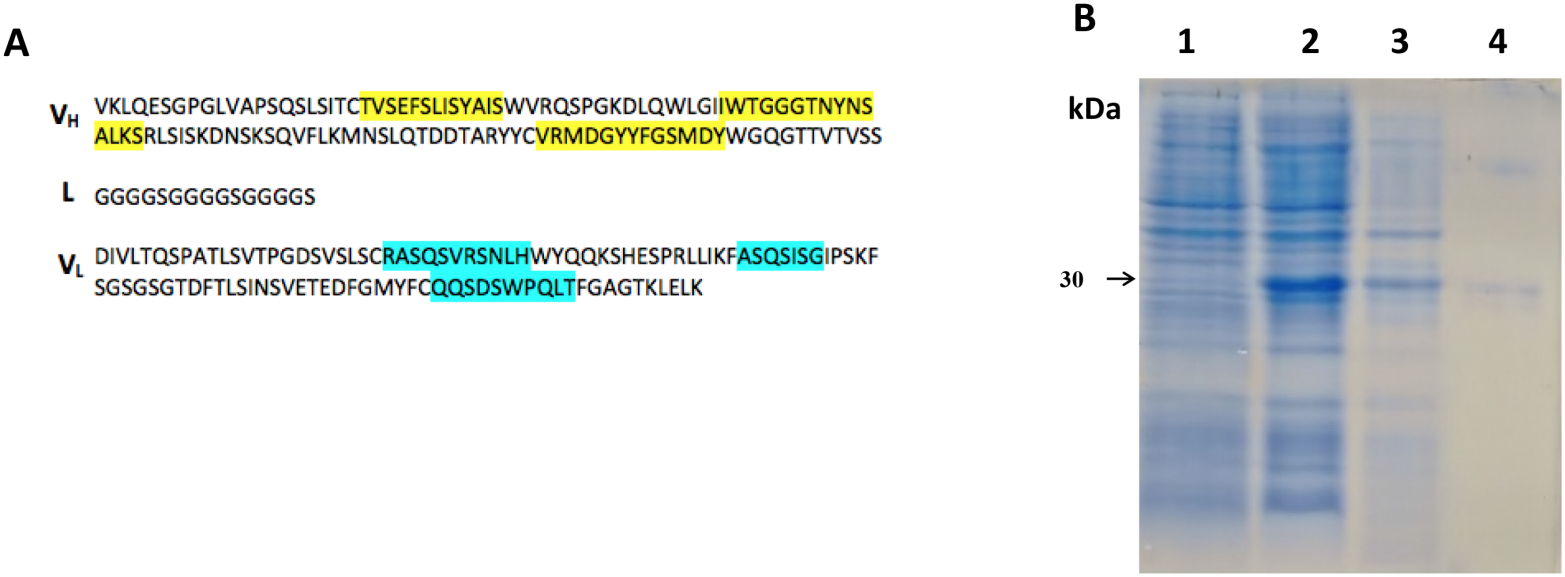
Construction, expression and purification of scFv-LT. (A) scFv-LT amino acids sequence: V_H_—Heavy chain variable domain, L—Linker and V_L_—Light chain variable domain sequence. CDRs are highlighted in yellow for V_H_ and in blue for V_L_. (B) SDS-PAGE analysis of scFv-LT recombinant antibody expression and purification. Lanes: **1.** C43(DE3) non-induced fraction; **2.** C43(DE3) induced fraction; **3.** Insoluble fraction; **4.** Purified scFv-LT.

**Fig 2:**
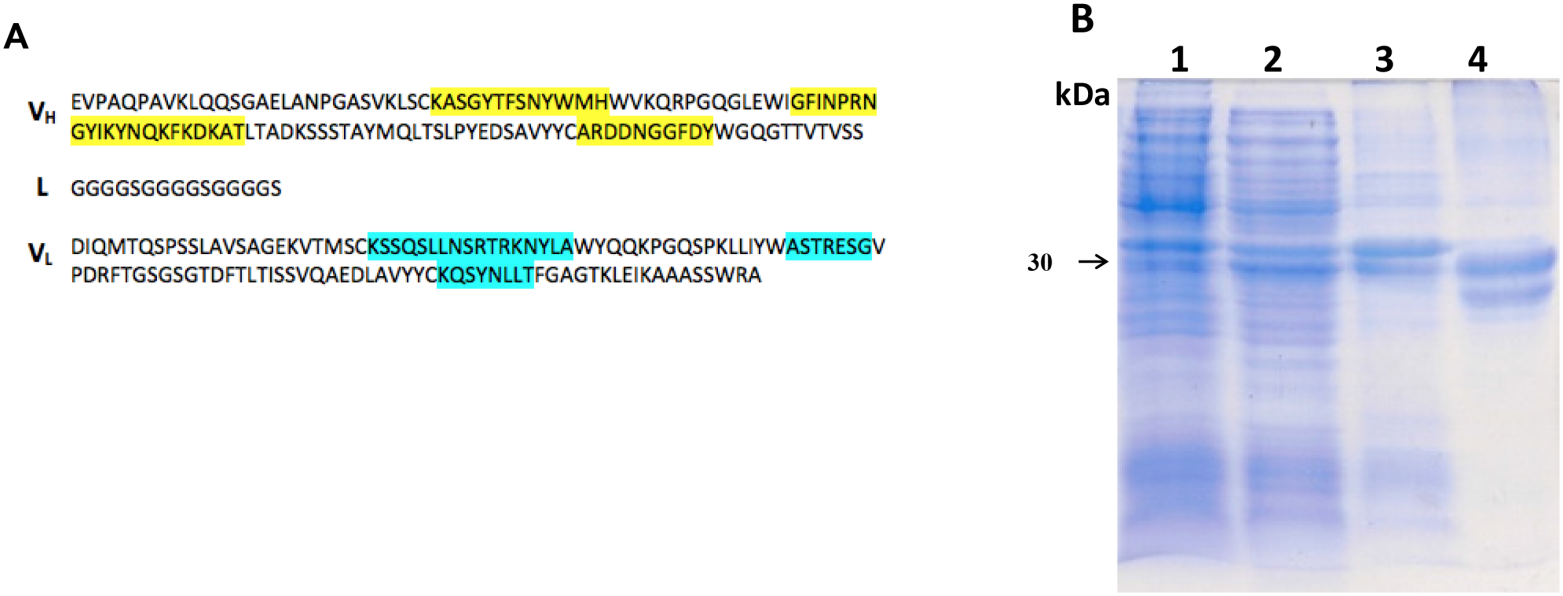
Construction, expression and purification of scFv-ST. (A) scFv-ST amino acids sequence: V_H_—Heavy chain variable domain, L—Linker and V_L_—Light chain variable domain sequences. CDRs are highlighted in yellow for V_H_ and in blue for V_L_. (B) SDS-PAGE analysis of scFv-ST recombinant antibody expression and purification. Lanes: **1.** BL21(DE3) non-induced fraction; **2.** BL21(DE3) induced fraction; **3.** Insoluble fraction; **4.** Purified scFv-ST.

DNA fragments of approximately 720 bp were obtained from synthetic genes (VH-linker-VL orientation) amplification corresponding to scFv-LT and scFv-ST. These fragments were subcloned into pET28a vector, confirmed by restriction analysis using *Bam*HI / *Xho*I, and sequencing, which showed that scFv-LT and scFv-ST fragments presented 100% identities with the corresponding synthetic genes, and the complementary domain regions (CDRs) sequences were preserved (Figs 1A and 2A).

### Expression and purification of scFv recombinant antibodies

Different bacterial hosts and culture media were tested in order to determine the best expression conditions of plasmids pET-scFv-LT and pET-scFV-ST (data not shown). *E*. *coli* C43(DE3) grown in Luria Bertani broth and *E*. *coli* BL21(DE3) grown in 2YT broth presented the best results regarding expression for scFv-LT and scFv-ST production, respectively (data not shown). Recombinant clones yield a concentration of 4,02 mg/L of scFv-LT and 1,53 mg/L of scFv-ST (Figs 1B and 2B). When compared to the not induced fractions (lanes 1), a band corresponding to scFv-LT (Fig 1B, **lane 2**) and scFv-ST (Fig 2B, **lane 2**) recombinant antibodies with apparent molecular weight of 30 kDa can be observed. Lanes 3 show scFv-LT (Fig 1B) and scFv-ST (Fig 2B) expressed as inclusion bodies, respectively; while lanes 4 allows the visualization of protein molecule corresponding to recombinant antibodies.

### Recombinant antibodies functionality

After purification both antibodies were refolded by stepwise dialysis. The refolding protocol employed was efficient since it was reproducible and both scFvs showed high stability under 4°C, -80°C and lyophilization storage, up to six months. Moreover, no detected change in relative molecular weight following treatment with high concentrations of urea was observed. Their reactivity was tested against LT and ST toxins by immunofluorescence, ELISA and immunoblotting.

**Fig 3:**
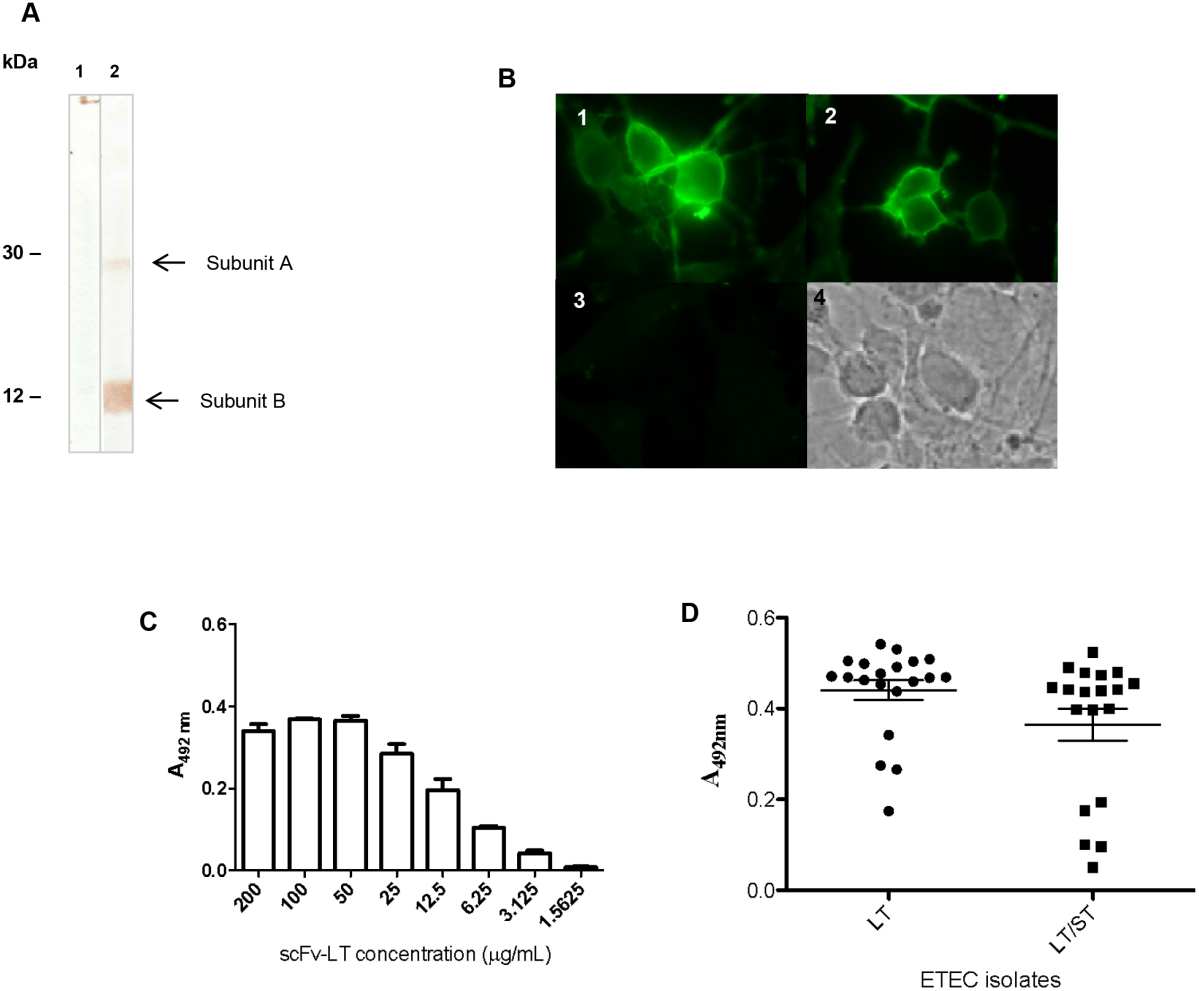
Reactivity of scFv-LT recombinant antibody against LT toxin. (A) Nitrocellulose membrane containing the LT toxin protein after SDS-PAGE 15% were subjected to immunoblotting with antibodies. Lanes: **1.** scFv-LT 200 μg/mL, **2.** 50 μg/mL anti-LT mAb. (B) Immunofluorescence assay after LT interaction with Y-1 cells. Panel 1. Reactivity with scFv-LT; Panel 2. Reactivity with anti-LT mAb. Panel 3. Reactivity with scFv-LT in the absence of LT toxin. Panel 4. Light microscopy. (C) scFv-LT titration with LT toxin by capture ELISA. (D) Detection of LT in culture supernatants of LT- and LT/ST-producing ETEC isolates by capture ELISA.

As LT toxin has two subunits, scFv-LT recombinant antibody was tested by immunoblotting to verify which subunit was recognized. Analyzing the immunoblotting on Fig 3A, we can observe that even in the higher concentration of scFv-LT used (200 μg/mL), the antibody was not able to recognize either A or B toxin subunits (**lane 1**), unlike the anti-LT monoclonal antibody (positive control), which was able to recognize both subunits (**lane 2**).

The reactivity of scFv-LT recombinant antibody against LT toxin was also tested by indirect immunofluorescence assay using Y1 cells. In Fig 3B, the fluorescent Y1 cells can be observed after incubation with scFv-LT (Fig 3B, **panel 1**) or mAb anti-LT (Fig 3B, **panel 2**). No fluorescence was observed when the cells were incubated without the toxin (Fig 3B, **panel 3**). Using a capture ELISA, scFv-LT was able to recognize LT toxin in a dose-dependent manner (Fig 3C). The scFv-LT antibody was also able to recognize only LTI-producing strains in culture supernatants of different ETEC isolates, since LT-negative strains were employed as control defining the cut-off in all capture ELISA experiments (Fig 3D).

**Fig 4:**
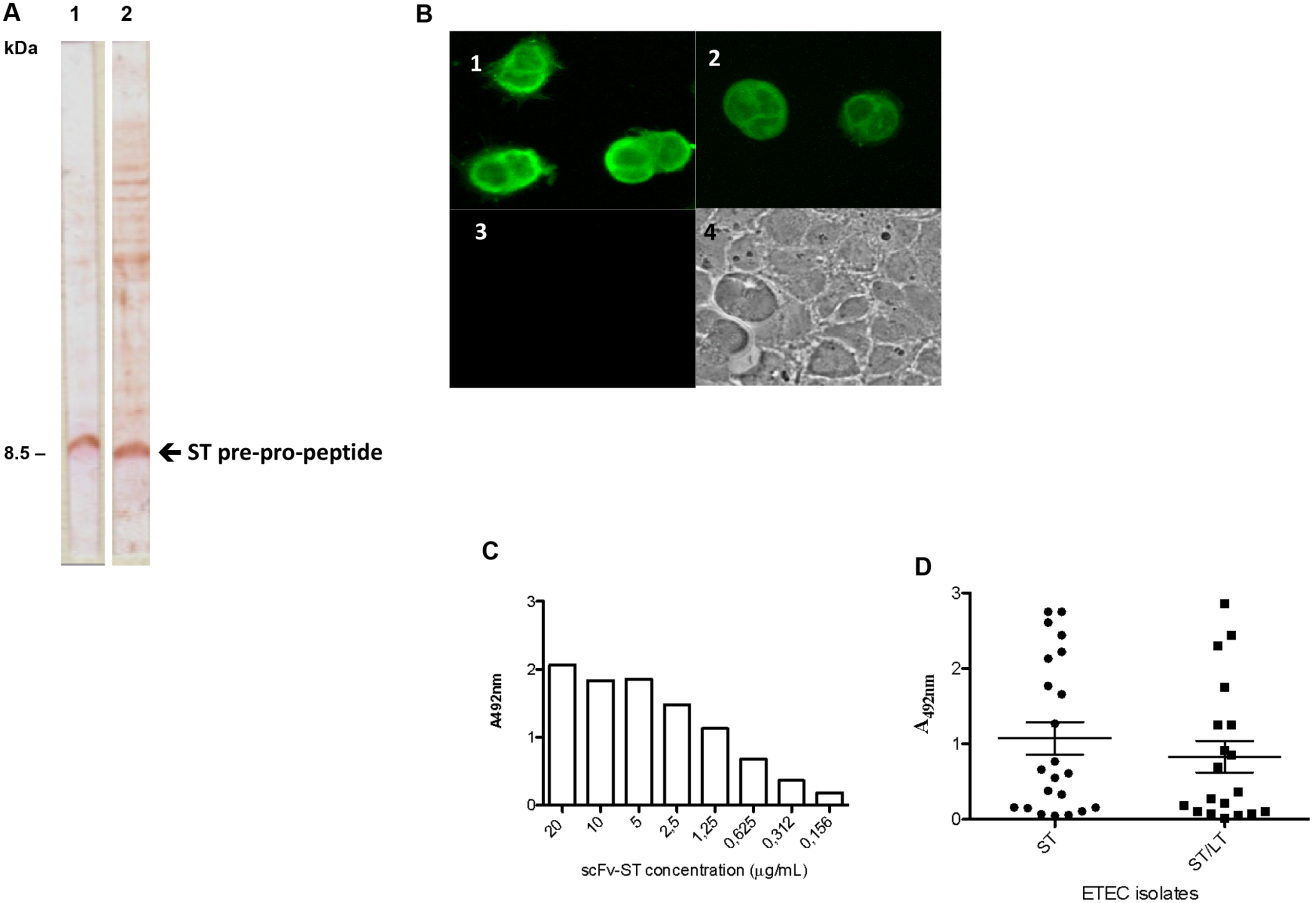
Reactivity of scFv-ST recombinant antibody against ST toxin. (A) PVDF membranes containing the bacterial lysates fractions after PAGE 10%/tricine gel was subjected to immunoblotting with antibodies. **1.** 30 μg/mL of scFv-ST. **2.** 30 μg/mL anti-ST mAb. (B) Immunofluorescence assay after ST interaction with Caco-2 cells. Panel 1. Reactivity with scFv-ST; Panel 2. Reactivity with anti-ST mAb. Panel 3. Reactivity with scFv-ST in the absence of ST toxin. Panel 4. Light microscopy. (C) scFv-ST titration with ST toxin by indirect ELISA. (D) Detection of ST in culture supernatants of ST- and LT/ST-producing ETEC isolates by indirect ELISA.

The scFv-ST reacted to pre-pro-peptide ST (Fig 4A, **lane 1**) as observed with anti-ST mAb (Fig 4A, **lane 2)**. On the other hand, when immunofluorescence assay using Caco-2 cells was employed, the intensity of recombinant antibody reactivity (Fig 4B, **panel 1**) was greater than the original mAb anti-ST (Fig 4B, **panel 2**). No fluorescence was observed when the cells were incubated without the toxin (Fig 4B, **panel 3**). Indirect ELISA with ST toxin resulted in a concentration dependent recognition of scFv-ST (Fig 4C). Also, the antibody demonstrated to be able to detect ST toxin secreted on culture supernatant of STh/STp-producing enterotoxigenic *E*. *coli* isolates, since there were no recognition of non-ST producing strains culture supernatant (Fig 4D).

## Discussion

The importance of ETEC in diarrheal disease in developing countries lead to the development of several immunoserological assays in the past [6]. But, unfortunately diagnosis of this pathogen is not included in minimally equipped laboratories, thus tools and/or methods that can be used to identify these organisms are not available [6, 7]. Recombinant antibodies, such as single chain fragment variable (scFv), rise as an alternative tool for that approach [32, 33]. Diverse scFvs molecules have been previously developed for antigens detection of different DEC pathotypes. In terms of ETEC, some authors described scFv against F5 and K99 colonization factors [34], as well as the LT toxin [35].

Menezes et al [32] developed the scFv-intimin, which recognizes both native intimin present in enteropathogenic *E*. *coli* (EPEC) prototype strain E2348/69 and recombinant intimin. This recombinant antibody is an excellent tool for diagnosis of EPEC and also enterohemorrhagic *E*. *coli* (EHEC) by immunofluorescence [33]. EspA and intimin proteins of EHEC O157:H7 were targets for the development of scFvs that recognize both antigens in O157 and O111 EHEC tested [36]. For STEC, some anti-Stx2 scFv were able to recognize and neutralize the Stx2 toxin [37, 38, 39, 40].

Among the various expression systems used for scFvs production, *e*.*g*. bacteria, mammalian cells, plants, yeast and insect cells, *E*. *coli* is the most used expression system for scFvs production due to its characteristics of easy handling, rapid and inexpensive growth, and high protein yield [41]. At the beginning of scFv development many research groups, including us, used the phagemid pCANTAB 5E vector [42], and the produced antibodies were sufficient for the selection of clones, but the yield was not sufficient for further characterization. The production approach used in the present work for scFv-LT and scFv-ST was the cloning of both fragments into pET28a vector from synthetic genes (VH-linker-VL orientation) for expression in *E*. *coli*. The scFv genes were optimized for expression in *E*. *coli*, in order to overcome codon usage bias from original sequence derived from hybridomas. The pET28a vector was selected since it has T7-based expression which increases the protein expression yield [43, 44, 45, 46]. Even though milligrams of either scFv-LT or scFv-ST were produced, they were expressed as inclusion bodies. The production of scFvs in soluble and active form in *E*. *coli* is difficult to obtain, since the reducing environment of the bacterial cytoplasm is not compatible with disulfide bonds formation, resulting in unstable molecules or even aggregates [47], moreover several studies have reported the same issue with antibody fragments cloned into pET28a vector [45, 48, 49, 50].

In order to obtain active scFv-LT and scFv-ST, the refolding methodology was adapted for each molecule after inclusion bodies solubilization and purification. The stepwise dialysis was employed for both recombinant antibody, since it has been successful used for scFv refolding [26, 33] and, the adoption of this method combined with the use of folding additives contributed to obtain active fragments. Folding additives, such as L-arginine and oxidized/reduced glutathione, are commonly used on recombinant fragments refolding, assist correct folding of molecules and avoid aggregation [27, 49, 51].

Regarding functional analysis, both scFvs were able to recognize their respective antigens. By capture ELISA, scFv-LT was able to recognize not only LT purified toxin but also LT toxin produced by 100% of all ETEC strains tested. However, when scFv-LT was tested by indirect ELISA or immunodot, the refolded scFv-LT was able to recognize only LT purified toxin, but not the toxin present in culture supernatant of H10407 strain (data not shown). Nevertheless, scFv-ST was efficient in indirect ELISA assays using low antibody concentration and by immunoblotting reacting with to ST pre-pro-peptide contained into culture extract.

Immunoserological assays have great advantages over other diagnostic methods due to their high specificity, sensitivity, and easy sample preparation, in addition to the rapid development, improving the detection of these strains in endemic settings. The results presented here suggest that both scFv-ST as scFv-LT can be useful tools for the development of diagnostic methods such as latex agglutination assay and/or immunochromatographic tests for ETEC detection, which might be used in clinical laboratories, epidemiological studies and control of outbreaks of these pathogenic bacteria.

Animal ETEC strains are known to produce enterotoxins similar to those of human strains. The LT from animal strains, designated LTI, is similar to the LT produced by human ETEC; however, another variety designated LTII is only found in animals and is not associated with clinical human disease. Also, animal strains produce two major types of ST, designated STa (STI) and STb (STII). As in humans, both STh (STIb) and STp (STIa) may be produced by animal strains [6]. Herein, both fragments of antibodies were capable to detect LTI and STIb and STIa from ETEC strains.

Besides, these antibodies fragments could be manipulated and engineered as different constructions such as scFvs fused to alkaline phosphatase, which may facilitate the immunodiagnostic making possible immunoassays be done directly, without the labeled secondary antibody, thereby reducing costs and time for obtaining results [52]. In conclusion, we successfully developed and characterized two recombinant antibodies produced in *E*. *coli* against LT (scFv-LT) and ST (scFv-ST) toxins of ETEC. These scFv were capable of detecting LT and ST in ETEC strains, being promising tools for ETEC diagnosis.
